# Optimization of Online Soluble Solids Content Detection Models for Apple Whole Fruit with Different Mode Spectra Combined with Spectral Correction and Model Fusion

**DOI:** 10.3390/foods13071037

**Published:** 2024-03-28

**Authors:** Yang Li, Yankun Peng, Yongyu Li, Tianzhen Yin, Bingwei Wang

**Affiliations:** College of Engineering, National R&D Center for Agro-Processing Equipment, China Agricultural University, 17 Qinghua East Road, Beijing 100083, China

**Keywords:** different spectral modes, apple whole fruit soluble solids content, online detection, spectral correction, model fusion

## Abstract

Soluble solids content (SSC) is one of the main quality indicators of apples, and it is important to improve the precision of online SSC detection of whole apple fruit. Therefore, the spectral pre-processing method of spectral-to-spectral ratio (S/S), as well as multiple characteristic wavelength member model fusion (MCMF) and characteristic wavelength and non-characteristic wavelength member model fusion (CNCMF) methods, were proposed for improving the detection performance of apple whole fruit SSC by diffuse reflection (DR), diffuse transmission (DT) and full transmission (FT) spectra. The modeling analysis showed that the S/S- partial least squares regression models for all three mode spectra had high prediction performance. After competitive adaptive reweighted sampling characteristic wavelength screening, the prediction performance of all three model spectra was improved. The particle swarm optimization–extreme learning machine models of MCMF and CNCMF had the most significant enhancement effect and could make all three mode spectra have high prediction performance. DR, DT, and FT spectra all had some prediction ability for apple whole fruit SSC, with FT spectra having the strongest prediction ability, followed by DT spectra. This study is of great significance and value for improving the accuracy of the online detection model of apple whole fruit SSC.

## 1. Introduction

Apple is one of the most important fruits in the world, which consumers love for its rich nutrients [1]. With the economic development and the improvement of living standards, consumers have higher and higher quality requirements for apples [2]. Soluble solids content (SSC), as one of the main internal quality indicators of apples, directly determines consumers’ willingness to buy and price [3]. The use of traditional physical and chemical test methods for apple SSC detection has the disadvantages of destroying samples, long detection time, and small detection sample size, which cannot meet the demand of batch testing [4]. In recent years, visible/near-infrared (Vis/NIR) spectroscopy technology has been widely used in the research field of internal quality detection of fruit due to its advantages of nondestructive, rapid, online detection and low cost [5].

For spectroscopic detection, the interaction of light with tissue can be described in terms of two fundamental processes related to absorption and scattering [6]. Absorption depends strongly on the chemical composition of the tissue while scattering is mainly caused by differences in physical properties (e.g., particle size and shape, sample packing, and sample surface) [7]. Scattering leads to two consequences; the first is the lengthening of the optical range, which introduces a multiplicative term. The second is photon loss, which would be incorrectly counted as absorption, thus introducing an additive term [8]. Thus, the light scattering effect consists of both additive and multiplicative effects [9]. The additive effect mainly leads to a baseline drift in the spectrum, while the multiplicative effect “scale” the entire spectrum [10,11]. Significant additive and multiplicative effects in spectral data may invalidate commonly used multivariate linear models [12]. Therefore, the key to quantitative spectral analysis is to eliminate the additive and multiplicative effects in the original spectra as much as possible and extract the spectral information that is linearly correlated with the target chemical components. Spectral pre-processing algorithms, such as multiple scattering correction (MSC), standard normal variational transform (SNV), and min–max normalization (NM), are common light-scattering correction algorithms that are widely used in spectral pre-processing [13]. Different spectral pre-processing algorithms may apply to different samples, so when quantitative modeling is carried out, different spectral pre-processing algorithms are usually compared to find the best spectral pre-processing algorithm applicable to that sample. For a naturally grown organism sample such as an apple, the physical property differences between samples are more significant, especially the morphology and size differences are also larger, which can lead to significant light range differences when spectra are collected. Therefore, for apple spectral correction, targeted elimination of multiplicative effects in the spectra may improve the quality of the apple spectra and, thus the predictive performance of the model.

When it comes to spectral acquisition, three common fruit spectral acquisition modes are diffuse reflection (DR), diffuse transmission (DT), and full transmission (FT) [14]. The characteristics of different spectral acquisition modes are not the same, resulting in different applicable scenarios. The DR mode of spectral acquisition has a simple structure and is suitable for collecting spectral information on the surface and shallow layers of apples, but it is easily affected by the specular reflection on the surface of the fruits, leading to a decrease in detection accuracy [15]. DR mode is generally used for SSC detection in some areas of fruit, but some scholars have used DR spectra for whole fruit SSC detection, which also has some predictive ability [16]. The DT mode can obtain more information about the internal spectrum of the fruit, avoiding the interference of specular reflection and shortening the optical range of transmitted light, but it is easily affected by stray light through the fruit and between the fruit holder. For the FT mode, the fruit is placed between the light source and the fiber optic probe so that the spectral information of the whole fruit can be collected and the light from the light source can be blocked entirely. However, when the intensity of the light source is weak, or the diameter of the fruit is large, the quality of the acquired spectra will be reduced [17]. The characteristics of different model spectra may lead to differences in model prediction performance when an online detection of apple whole fruit SSC is performed.

Model optimization through variable selection is also key to building a simple, fast, and robust predictive model, as modern spectroscopic instruments often have high resolution, and the resulting spectra include thousands of variables [18]. Too much spectral data has at least two drawbacks: firstly, the calibration and implementation of the model is very time-consuming, which inevitably affects the ability of the model to perform fast analyses online, and secondly, some of the spectral variables in the full spectra are irrelevant and redundant, which reduces the predictive power of the model. However, since each variable selection method is data-based and has its principles, advantages, disadvantages, and applications, no study has shown which method is optimal [19]. The optimal characteristic wavelength screening algorithms for different mode spectra may differ and must be studied and analyzed.

Partial least squares regression (PLSR) is a commonly used modeling method in spectral analysis. The method finds potential variables that can be effectively used to explain concentration variations using both spectral data and the concentration of the sample. In addition to its simplicity and computational efficiency, PLSR gives better results than other multivariate methods, such as multiple linear regression (MLR) and principal component regression (PCR). Currently, nonlinear model-building methods, such as the least squares support vector regression (LS-SVR) and particle swarm optimization–extreme learning machine (PSO-ELM) algorithms, have been widely used in modeling for quantitative spectral analysis [20]. Due to the different spectral acquisition methods and data types, there may be differences in the modeling results using linear and nonlinear modeling methods. Therefore, it is necessary to explore the best modeling methods applicable to different modes of spectra.

Many studies have been conducted to investigate and optimize the spectral pre-processing algorithms, characteristic wavelength screening algorithms, modeling methods, etc., and to establish an optimal prediction model [21]. A single model may have problems such as poor robustness and generalization ability, which will limit the further improvement of model accuracy [22]. A model fusion modeling strategy has been proposed to further improve the model performance [23,24]. Model fusion is not a specific algorithm but an idea of merging multiple weak models into a strong model. In the past, when using characteristic wavelength modeling, the best characteristic wavelength prediction model was identified through comparative analysis. However, other characteristic wavelength models with relatively poor results would be discarded. This not only consumes the time and effort of model building but also ignores the possible contribution of other characteristic wavelength prediction models to the prediction results. In addition, spectral information other than characteristic wavelengths is discarded when modeling with characteristic wavelengths. However, non-characteristic wavelength data may also contain information that is often ignored and weakly correlated with the components. Therefore, multiple characteristic wavelength member model fusion (MCMF), as well as characteristic wavelength non-characteristic wavelength member model fusion (CNCMF) approaches may be able to fully utilize the contribution of the discarded predictive models and wavelength variables to the prediction results, thus further improving the predictive performance of the models.

Aiming at the above problems, the main contents of this study include the following aspects:

(1) To explore spectral pre-processing algorithms applicable to apple to improve spectral quality;
(2) To explore the effects of different mode spectra (DR, DT, and FT), spectral pre-processing algorithms, characteristic wavelength screening algorithms, and modeling methods on the on-line detection model of SSC for whole apple fruit;
(3) To explore the effect of model fusion methods on improving model prediction performance.

## 2. Materials and Methods

### 2.1. Spectral Acquisition Devices and Acquisition Methods

**Figure 1:**
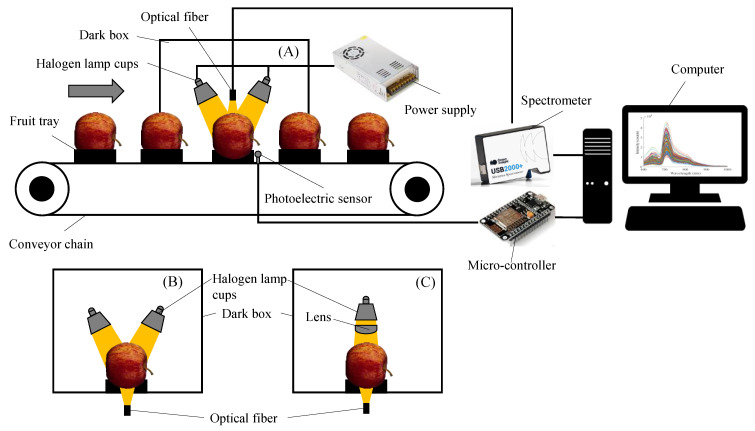
Spectral online acquisition devices. (**A**) Diffuse reflection spectral acquisition device; (**B**) diffuse transmission spectral acquisition device; and (**C**) full transmission spectral acquisition device.

In this study, online spectral acquisition devices for DR, DT, and FT spectra were used to dynamically collect spectral information of apples. The DR spectral acquisition device (Figure 1A) consisted of an optical fiber (Vis/NIR, Ocean Optics, Dunedin, FL, USA), a spectrometer (USB2000+, Ocean Optics, Dunedin, FL, USA), four 35 W halogen lamp cups (ESS MR 11 35 W, Philips, Amsterdam, The Netherlands), a micro-controller (ESP8266, TW, ShenZhen, China), an opposing photoelectric sensor (CTD-1500P, OPTEX, Kyoto, Japan), a power supply (S-350-120, Li-Cheng-An, Shenzhen, China), a conveyor chain, fruit trays, a computer, and a dark box. The device collected the DR spectral information of apples through the optical fiber at the upper end.

Figure 1B shows the DT spectral acquisition device, similar to the DR spectrum acquisition device but differing in the spectral acquisition part. The device collected the spectral information of the transmitted apple through the optical fiber at the lower end. 

Figure 1C shows the FT spectral acquisition device. The device used a 100 W halogen lamp cup (6834FO, Philips, Amsterdam, The Netherlands) as the light source, and a lens (with a focal length of 40 mm) was mounted in front of the light source to avoid too much dispersion of the light emitted from the light source. The device collected spectral information through the apple through an optical fiber at the lower end.

The control program of the device was developed based on PyQt and Arduino IDE. 

Before the spectral acquisition, the light source should be warmed up for 30 min to make the system reach a stable state. The integration time was set to 1 ms for the acquisition of the DR spectrum, 30 ms for the acquisition of the DT spectrum, and 200 ms for the acquisition of the FT spectrum. During spectral acquisition, transmission speed was set to 0.2 m/s, the conveyor chain was switched on, and the apples were placed horizontally on the fruit trays in the manner shown in Figure 1. When the apples reached the spectral acquisition position, the photoelectric sensor detected the position information and sent the in-place information through the micro-controller to the upper computer program, and the upper computer triggered the spectrum acquisition. For each sample, spectral information was collected three times, including DR, DT, and FT spectral information once each. Due to the differences in the noise range of the spectra collected by different spectral acquisition modes, the spectra within the range of 650–1000 nm were selected for the DR mode, and the spectra within the range of 600–900 nm were selected for the DT and FT modes. 

A polytetrafluoroethylene (PTFE) reference sphere of 80 mm diameter was used to collect the white reference. The dark reference was collected with the light source turned off. The absorbance was calculated using Equation (1) and used for subsequent modeling analysis.

(1) $${A=\log}_{10}\frac{1}{TR}=\log_{10}\frac{T_{white}-T_{dark}}{T_{raw}-T_{dark}}$$

where *A* is absorbance; TR is transmittance or reflectance; $T_{raw}$ is sample spectral intensity; $T_{white}$ is white reference spectral intensity; $T_{dark}$ is dark reference spectral intensity.

After the spectral acquisition, spectral pre-processing, characteristic wavelength screening, and modeling analysis were performed using MATLAB (R2016a; The MathWorks, Natick, MA, USA).

### 2.2. Preparation of Samples

In this study, Fuji apples were used as the research object to establish the online detection models of apple SSC. The Fuji apples were grown in Yantai City, Shandong Province, and 105 apples without mechanical damage and external defects were selected and transported to the nondestructive techniques laboratory in the College of Engineering of China Agricultural University. The surface of the apple samples was wiped clean, numbered, and stored at 4 °C. Before the spectral acquisition, the apples were placed at room temperature (20 °C) for 24 h to minimize the effect of temperature variation on spectral acquisition. Before modeling, the samples must be divided into correction and prediction sets. This study used a randomized grouping method to divide the samples into a correction set and a prediction set at a ratio of approximately 3:1.

### 2.3. SSC Measurement

The SSC of apples was determined using a refractometer (PAL-BX/AC5, ATAGO Co., Ltd., Tokyo, Japan) in conjunction with destructive methods. The SSC measurement range of the refractometer is 0.0–60.0%, with a resolution of 0.1% and an accuracy of ±0.2%. After collecting the spectra, the juice of the whole apple was extracted using a juicer, poured into a beaker, and stirred well, and the apple SSC was determined by dropping the juice into the refractometer measuring position using a rubber-tipped burette. Each sample was collected three times, and the average value was taken as the SSC of that sample.

### 2.4. Spectral Scattering Correction Method

For non-homogeneous mixtures such as apples, the relationship between the raw absorption spectra and the content of the target chemical components is shown in Equation (2) [11,25,26]:

(2) $$X_{i}=p_{i}\sum_{j=1}^{J}c_{i,j}s_{j}+b_{i}1$$

where **X***_i_* is the absorption spectrum vector of the *i*th mixture sample; *p_i_* is the multiplication factor, which represents the multiplicative effect of the change in effective optical range due to the change in physical properties of the sample on the spectrum of the *i*th mixture sample; *c_i,j_* is the concentration of the chemical component in section *j* of the *i*th sample; **s***_j_* is used to evaluate the light absorption capacity of the *j*th chemical component, which is mainly related to the type of chemical component; *b_i_* is an addition coefficient that represents the baseline of the spectrum, mainly related to the environment and sample state; **1** is a row vector with element 1.

From Equation (2), it can be seen that *p_i_* and *b_i_* are sample-dependent variables, resulting in the original spectra no longer showing a regular linear law with the target chemical content. Therefore, eliminating *p_i_* and *b_i_* is the key to ensuring the robustness of the multiple regression model.

#### 2.4.1. Addition Coefficient Elimination

Some scholars have proposed the linear regression correction (LRC) method, in which the intercept is obtained by constructing a one-dimensional linear regression equation between the sample spectrum and the average spectrum, and the intercept is subtracted from the original spectrum to achieve the elimination of *b_i_* [12]. This method is equivalent to a simplified version of the MSC algorithm, which eliminates only the additive coefficients in the spectrum. After the elimination of *b_i_*, Equation (2) can be changed to Equation (3).

(3) $$X_{c,i}=p_{i}\sum_{j=1}^{J}c_{i,j}s_{j}$$

where X*_c_*_,*i*_ is a vector of absorption spectra for the *i*th mixture sample affected only by multiplicative effects.

#### 2.4.2. Multiplication Coefficient Elimination

The elimination of multiplicative coefficients can be achieved by dividing the spectral data X*_c_*_,*i*_ after the elimination of additive coefficients by the spectral data x*_i_*_,λ_ in which the wavelength is λ, as shown in Equation (4).

(4) $$X_{s/s,i}=\frac{X_{c,i}}{x_{i,\lambda }}=\frac{p_{i}\sum_{j=1}^{J}c_{i,j}s_{j}}{p_{i}c_{i,\lambda }s_{\lambda }}=\frac{\sum_{j=1}^{J}c_{i,j}s_{j}}{c_{i,\lambda }s_{\lambda }}$$

where X_s/s,*i*_ is the vector of absorption spectra of the *i*th mixture sample after correction for spectra-to-spectra ratio (S/S); *c_i_*_,λ_ is the concentration of the substance reflected by the wavelength λ; s_λ_ is the extinction coefficient of the substance reflected by wavelength λ.

From Equation (4), when *c_i_*_,λ_s_λ_ is a constant value that is not sample-dependent, the spectral data show a better linear relationship with the target chemical composition content. Assuming that the chemical composition content represented by wavelength λ in the spectra varies less for each sample, *c_i_*_,λ_s_λ_ can be approximated as a constant value at this time. The spectral correction can be completed by substituting this wavelength spectral data into Equation (4). This study adopts the global search method, substituting the spectral data at each wavelength into Equation (4) in turn for correction, and then constructs the PLSR prediction models of the corrected spectra with the content of the target components by Monte Carlo cross-validation method with the root mean square of the standard error of cross-validation (RMSECV) was minimized as a criterion to determine this wavelength data.

In summary, the S/S spectral correction method proposed in this study achieves spectral scattering correction by first eliminating the additive coefficients of the original spectra and then eliminating the multiplicative coefficients. The elimination of the multiplicative coefficients is oriented to the optimal model prediction results, highlighting the effect of the elimination of the multiplicative coefficients on enhancing the model prediction performance. This method was used to correct the spectra in subsequent studies, and the modeling results were used to judge the correction effect.

### 2.5. Spectra Pre-Processing Methods

Pre-processing was performed to remove the variations in the spectrum due to disturbances and to highlight the components related to SSC. Spectral pre-processing methods such as MSC, SNV, and NM are most widely used in spectral pre-processing. Therefore, this study used MSC, SNV, NM, and S/S to pre-process the spectra and develop prediction models for apple SSC. A comparative analysis of the modeling results would verify the effectiveness of the proposed spectral pre-processing method.

### 2.6. Characteristic Wavelength Screening Methods

Since the full spectrum contains much irrelevant and collinear information, it affects the prediction model’s performance. Therefore, characteristic wavelength screening algorithms were used to select wavelength points in the spectrum that were closely related to the SSC information, which could reduce the number of spectral variables and improve the model prediction performance. In this study, the competitive adaptive reweighted sampling (CARS), bootstrapping soft shrinkage (BOSS), and interval variable iterative space shrinkage approach (iVISSA) algorithms were used to screen characteristic wavelength.

CARS is an algorithm used in conjunction with the regression coefficients in PLSR to screen wavelength variables in a spectrum. Firstly, a part of the calibration set of the sample is randomly selected for PLSR modeling, the random modeling is repeated several times, and the exponentially decreasing function (EDP) is used to remove the wavelength points with smaller weights of the regression coefficient values [27]. After several modeling sessions, the wavelength points with larger weights of absolute values of regression coefficients are screened out to construct a subset of variables, and the resulting new subset of variables is then subjected to PLSR modeling and analysis, in which the subset with the smallest RMSECV is the optimal combination of wavelength variables selected. The parameters used for CARS characteristic wavelength screening in this research were as follows: the maximum number of latent variables (LVs) was set to 15, five-fold cross-validation, and 100 sampling runs.

The BOSS algorithm is derived from the idea of weighted bootstrap sampling (WBS) and model population analysis (MPA) [28]. The weights of the variables are determined based on the absolute values of the regression coefficients, WBS generates sub-models based on the weights, and MPA is used to analyze the sub-models to update the variable weights. The optimization process follows the “soft shrinkage” rule, i.e., smaller weights are assigned instead of directly eliminating unimportant variables. The algorithm runs iteratively until the number of variables reaches one. The set of variables with the smallest RMSECV is selected as the result of feature wavelength screening. The parameters used for BOSS characteristic wavelength screening in this research were as follows: the maximum LVs were set to 15, five-fold cross-validation, and 1000 sampling runs.

iVISSA is a wavelength interval selection algorithm proposed by Deng et al. based on the variable iterative space shrinkage approach (VISSA) [29]. The algorithm combines global and local search to intelligently and iteratively optimize the position, width, and combination of wavelength intervals. In the global search process, the advantages of VISSA soft shrinkage are inherited to search for the positions and combinations of informative wavelengths, while in the local search process, the continuity information of the spectral data is utilized to determine the widths of the wavelength intervals. The global and local searches are performed alternately for wavelength interval selection. The parameters used for iVISSA characteristic wavelength screening in this research were as follows: the maximum LVs were set to 15, five-fold cross-validation, and 500 sampling runs.

### 2.7. Model Fusion Methods 

**Figure 2:**
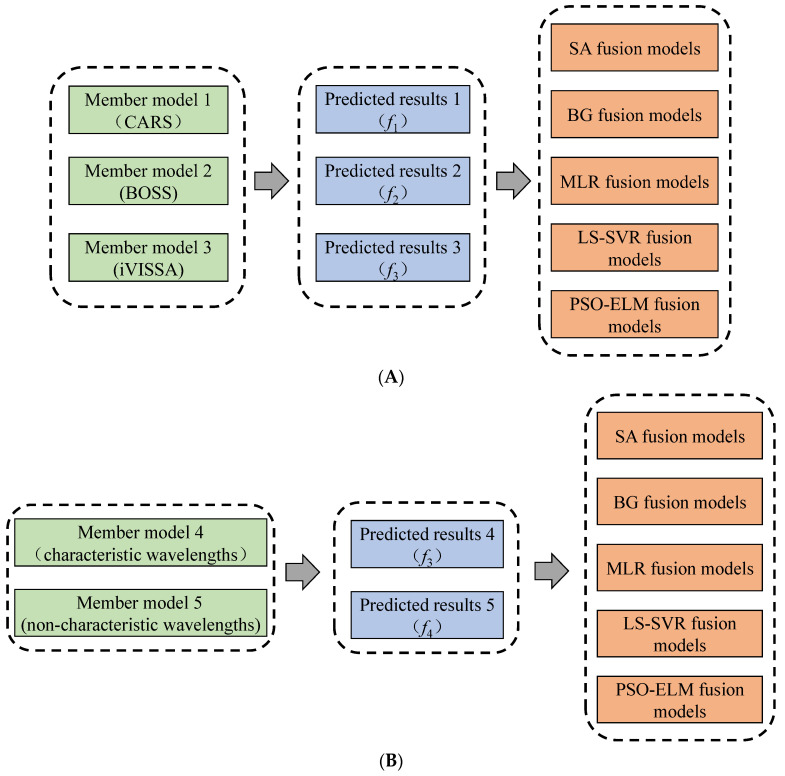
Model fusion methods. (**A**) Multiple characteristic wavelength member model fusion methods; (**B**) characteristic wavelength and non-characteristic wavelength member model fusion methods.

Model fusion is the process of fusing multiple weak models into one strong model. This method has the effect of collective decision-making, which can compensate for the error of a single model and further improve the model’s performance [30]. This study used two model fusion methods, multiple characteristic wavelength member model fusion (MCMF), and characteristic wavelength and non-characteristic wavelength member model fusion (CNCMF), to further optimize the prediction model for a single mode spectrum. Figure 2A shows the MCMF fusion methods, and Figure 2B shows the CNCMF fusion methods.

### 2.8. Modeling Methods

In this study, the models were divided into two categories, namely, single-mode spectral prediction models and fusion prediction models. Due to the large number of single-mode spectral variables, PLSR, LS-SVR, and PSO-ELM were used to build prediction models. For the fusion models, simple averaging (SA), Bates–Granger averaging (BG), MLR, LS-SVR, and PSO-ELM were used to build prediction models.

The above modeling methods are common modeling methods used in data analysis. SA averages the predictions of the member models as fusion predictions, which is equivalent to assigning the same weight to each model. BG assigns weights to the integrated model based on the associated variance [31]. For example, sensor predictions with higher predictive variance are assigned lower weights than sensor predictions with lower predictive variance. MLR is commonly used to construct linear relationships between multiple independent and dependent variables [32]. PLSR, as a multivariate regression analysis method, can perform downscaling and integrative screening of spectral data and analyze the correlation between two sets of variables, etc., and has high modeling stability [33]. The number of LVs in the PLSR model was selected using RMSECV results. 

LS-SVR is an improvement of the classical support vector machine, which is a powerful machine learning method in classification problems and pattern recognition [34]. The algorithm converts dot product operations in high-dimensional feature space into primitive spatial kernel functions. In the LS-SVR model of spectra, the radial basis function (RBF) is usually chosen as the kernel function for data analysis, which is adaptively stable and robust to the nonlinear modeling process of spectra. The two main parameters of the RBF are the regularization parameter (*γ*) and the width parameter (*σ*^2^). Different values of these two parameters lead to changes in the stability and predictive performance of the model [35]. Therefore, there is an urgent need to find optimization methods to optimize γ and σ^2^ to improve LS-SVR’s learning ability and generalization. In this study, the coupled simulated annealing (CSA) algorithm, grid search, and ten-fold cross-validation methods built into the least squares support vector machine (LS-SVM) toolbox (LS-SVM v 1.7, Suykens, Leuven, Belgium) were used to seek the optimal *γ* and *σ*^2^.

PSO-ELM is a method for optimizing ELM models based on a particle swarm optimization algorithm [36]. In PSO-ELM, the PSO algorithm is used to optimize the weights and biases of the implicit layer neurons in the ELM to minimize the prediction error. This can improve the prediction accuracy and generalization ability of ELM and avoid overfitting ELM models.

### 2.9. Model Evaluation Methods

The models were evaluated based on the correlation coefficient of calibration (*R*_c_), root mean square error of calibration (RMSEC), the correlation coefficient of prediction (*R*_p_), root mean square error of prediction (RMSEP), and relative percentage difference (RPD). For the same sample set, the larger *R*_c_, *R*_p,_ and RPD are, and the smaller RMSEC and RMSEP are, the better the predictive performance of the corresponding model. For different sample sets, it is more objective to use RPD to evaluate the predictive performance of the model. When RPD > 2, it indicates that the prediction effect is better, the prediction accuracy is high, and the established model can be used for actual detection. When 1.4 < RPD < 2, it indicates that the model prediction ability is ordinary, and the prediction accuracy needs to be improved. When RPD < 1.4, it indicates that the model prediction performance is poor and cannot be used for quantitative detection [37].

## 3. Results and Discussion

### 3.1. Analysis of Apple Spectra

**Figure 3:**
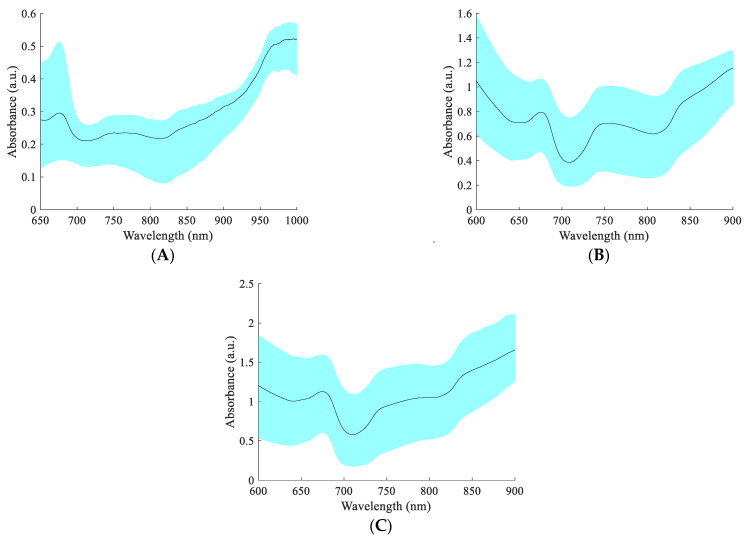
The spectra of apples. (**A**) Diffuse reflection spectra; (**B**) diffuse transmission spectra; and (**C**) full transmission spectra.

The spectra of 105 apples were dynamically collected using the spectral acquisition devices and methods in Section 2.1, and the absorbance was calculated according to Equation (1), as shown in Figure 3. The 650–700 nm visible light band in the figure is associated with pigments (e.g., chlorophyll and anthocyanins) in apple pericarp [4]. The 700–900 nm spectral range is associated with the C-H, O-H, and NH_2_ vibrations, where the C-H and O-H vibrations are closely related to the SSC [38,39]. The DR, DT, and FT spectra exhibited different absorbance values, with the absorbance of the transmission spectrum being higher than that of the DR spectrum. This is mainly because less light is transmitted through the apple, resulting in a lower intensity of light received by the fiber. The positions of the peaks and troughs of the DR, DT, and FT spectra had some similarities, but the shapes of the spectra had significant differences. The difference in spectral shape may be caused by the different sensitivity of different spectral acquisition methods to different wavelengths of light. Therefore, there may be differences in the ability of different spectral acquisition methods to predict the SSC of whole apple fruit.

### 3.2. Statistics of SSC

The SSC data of 105 apples were determined using the method in Section 2.3, as shown in Table 1.

As can be seen from Table 1, the SSC distributions of the samples in the correction set and the prediction set were more similar, and the correction set contained the SSC range of the prediction set. Therefore, the division of the calibration set and prediction set is reasonable, which is conducive to constructing more robust prediction models.

### 3.3. Model Results

This study developed the PLSR, LS-SVR, and PSO-ELM prediction models of apple SSC after spectral processing using MSC, SNV, NM, and S/S pre-processing algorithms. For the PLSR model, the Monte Carlo cross-validation method was used in this study to calculate the variation of RMSECV with the number of LVs, and the number of LVs was selected according to the minimum RMSECV principle [40]. For the LS-SVR model, this study first calculated the initial values of the parameters *γ* and *σ*^2^ by CSA, then constructed the grid based on the initial values, and finally fine-tunes the parameters by using grid search and ten-fold cross-validation methods to realize the optimization search for the parameters *γ* and *σ*^2^. The PSO method was used to optimize the initial weights and biases of the ELM model. The apple SSC modeling results based on the best pre-processing method are shown in Table 2.

As can be seen from Table 2, for the PLSR model, the S/S pre-processing spectra had the best modeling effect. This indicates that the S/S pre-processing method can eliminate the scattering effect in the spectra to a certain extent, improving the linear relationship between the spectral data and the SSC. The S/S algorithm is better than other pre-processing algorithms in correcting the scattering effect in the apple spectra. The modeling results can also show that the S/S algorithm has good generality and can be applied to the correction of DR, DT, and FT spectra simultaneously. For the LS-SVR model, the raw spectra had the best prediction performance. This may be caused by the fact that the raw spectra contain a lot of nonlinear information related to SSC. After the spectra were corrected using different pre-processing methods, the modeling effectiveness of the nonlinear modeling approach decreased. This may be caused by pre-processing algorithms that make the spectral data more linear in relation to the SSC. The SNV pre-processed spectra had the best predictive performance for the PSO-ELM model. Among all models, the S/S-PLSR model with three-mode spectra had the best prediction performance, followed by the SNV-PSO-ELM model.

All three mode spectra have some predictive ability for whole fruit SSC of apples, with FT spectra having the best predictive ability, followed by DT spectra. The reason is that the FT spectrum collects information on the whole apple and corresponds closely to the whole fruit SSC. DT spectrum can also reflect information from more regions of the apple, and its correspondence with SSC is only second to that of the FT spectrum. The modeling results show that the DR spectra can also predict the whole fruit SSC, which the correlation between the SSC of some regions of a single apple and the whole fruit SSC may cause. Mo et al. (2017) [41] classified a single apple into 29, 9, and 5 regions of interest and measured their SSC values separately. The results of the SSC analysis of 25 apples showed that for individual apples, the coefficient of variation in SSC between the 5 ROIs was the smallest, which was below 6.00%. It indicates a certain correlation between the SSC of some regions of a single apple and the average SSC of the whole apple, which is also the fundamental reason leading to the feasibility of predicting the SSC of the whole apple by DR spectroscopy. 

### 3.4. Model Results Based on Characteristic Wavelength

To eliminate the co-linear information and noise in the spectra, simplify the model, and improve the model prediction performance [19]. This study used the CARS, BOSS, and iVISSA algorithms to screen the wavelength data closely related to apple SSC and optimize the S/S-PLSR models for the three mode spectra.

**Figure 4:**
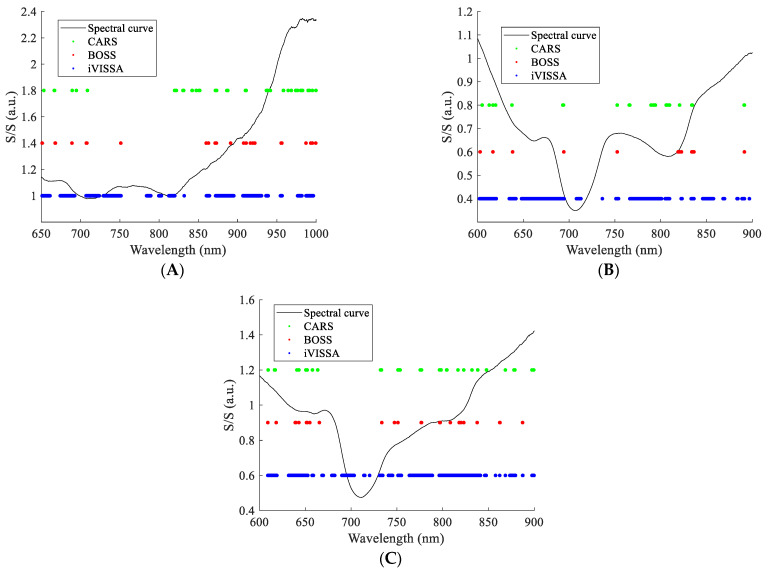
Characteristic wavelength screened by different algorithms. (**A**) DR spectral characteristic wavelengths; (**B**) DT spectral characteristic wavelengths; and (**C**) FT spectral characteristic wavelengths.

The results of characteristic wavelength screening are shown in Figure 4. As can be seen from the figure, the number of characteristic wavelengths screened by CARS and BOSS was relatively close, and the wavelength points had a high degree of overlap. iVISSA algorithm screened a larger number of characteristic wavelengths, which include the characteristic wavelengths screened by CARS and BOSS algorithms. The number of characteristic wavelengths screened in the NIR band was larger than the number of characteristic wavelengths screened in the Vis band. For apple SSC detection, the contribution of the NIR band is larger than that of the Vis band. The characteristic wavelengths screened by the three algorithms cover the range of wavelengths relevant to SSC.

The screened characteristic wavelengths were used to build prediction models for apple SSC, and the results are shown in Table 3. 

As seen in Table 3, the characteristic wavelength modeling results screened by the CARS algorithm were better than the full spectrum modeling results. This indicates that CARS characteristic wavelength screening can effectively eliminate irrelevant and covariant information in the original spectra and improve the prediction performance of the apple SSC model. Among the three spectra, the FT spectrum had the best modeling results, followed by the DT spectrum, and the DR spectrum had the worst modeling results. The reason is the strength of the correspondence between spectra and SSC. The RPD values of the modeling results of the three spectra after CARS characteristic wavelength screening were greater than 2, which indicates that all three spectra have high prediction performance for apple SSC after CARS characteristic wavelength screening. The modeling results of the screened characteristic wavelengths of the BOSS and iVISSA algorithms were decreased compared to those of the full spectra, which is probably because the modeling results of the BOSS and iVISSA algorithms eliminate the irrelevant and covariance information along with the elimination of characteristic wavelength data related to apple SSC. iVISSA algorithm screened the largest number of characteristic wavelengths, and although many wavelengths related to apple SSC were retained, some irrelevant and covariance information was also retained. Overall, the S/S-CARS-PLSR model predicted apple SSC best.

### 3.5. Model Fusion Results

#### 3.5.1. MCMF Modeling Results

To further improve the prediction performance of different mode spectra for apple SSC. The MCMF methods proposed in Section 2.7 were used to construct the prediction models for apple SSC, and the results are shown in Table 4.

As can be seen from Table 4, for the DR spectrum, the SA, BG, MLR, and PSO-ELM models of MCMF could further improve the prediction performance, while the LS-SVR model decreased the prediction performance; for the DT spectrum, the BG, MLR, and PSO-ELM models of MCMF could further improve the prediction performance, while the SA and LS-SVR models decreased the prediction performance; for FT spectra, the MLR, LS-SVR, and PSO-ELM models of MCMF were able to further improve the prediction performance, while the SA and BG models reduced the prediction performance; among all the fusion methods, the PSO-ELM model of MCMF had the greatest enhancement effect, followed by MLR; and among all the fusion models, the PSO-ELM model for FT spectra had the best prediction of the apple whole fruit SSC had the best prediction performance, followed by DT spectra. The PSO-ELM model of MCMF resulted in a fairly high prediction performance for the DR spectrum, which originally had a poor prediction performance, with the RPD increasing from 2.097 to 2.795. This method also increased the RPD for the DT spectra from 2.386 to 2.902 and the FT spectra from 2.703 to 3.461. From the weighting coefficients of the MLR model, it can be seen that each member model has a certain contribution to the prediction results, and the magnitude of the weighting coefficients is positively correlated with the prediction performance of the member models, and the better the prediction performance of the member models, the larger the weighting coefficients. The predictive performance of the fusion model may be correlated with the predictive performance of the member models, and the better the predictive performance of the member models, the better the predictive performance of the fusion model usually is. The fusion model can make full use of the predictive capability of each member model, thus improving the predictive performance of the model, and does not superimpose the covariance or noise information between different data. The MLR and PSO-ELM models of MCMF can further improve the predictive performance of the three-mode spectral model based on the traditional single model.

#### 3.5.2. CNCMF Modeling Results

From previous studies, it is known that the prediction performance of the fusion model is positively correlated with the prediction performance of the member models. Therefore, this study fused the characteristic wavelength and non-characteristic wavelength prediction models screened by the CARS algorithm. The characteristic wavelengths screened by the CARS algorithm were removed, and then the PLSR prediction models for non-characteristic wavelengths were established. The results are shown in Table 5.

From Table 5, it can be seen that the performance of the non-characteristic wavelength prediction models decreased compared to the characteristic wavelength, but the non-characteristic wavelength prediction models also had some prediction ability. It indicates that the non-characteristic wavelength also contains information related to apple SSC. Previous modeling methods using characteristic wavelengths did not make full use of the information related to apple SSC in the spectra. Therefore, using the fusion method of characteristic wavelength and non-characteristic wavelength member models can make full use of the contribution of the non-characteristic wavelength model to the prediction results. CNCMF modeling results, as shown in Table 6.

As can be seen from Table 6, for the DR spectrum, the SA, BG, MLR, LS-SVR, and PSO-ELM models of CNCMF could further improve the prediction performance; for the DT spectrum, only the PSO-ELM model of CNCMF improved the prediction performance, while all others decrease; for the FT spectrum, the LS-SVR and PSO-ELM models of CNCMF could further improve the prediction performance, while the SA, BG, and MLR models degrade the prediction performance. The PSO-ELM model of CNCMF significantly improved the prediction models of DR, DT, and FT spectra and slightly outperformed the PSO-ELM model of MCMF. However, the difference in the prediction performance of the two methods may be caused by the randomness in optimizing the PSO-ELM model parameters. Therefore, it can be considered that the prediction performance of the two prediction models is relatively close, and both can significantly improve the prediction performance of the models.

### 3.6. Discussion

The effectiveness of the proposed S/S algorithm for spectral correction is demonstrated by the results of PLSR modeling of DR, DT, and FT spectra. It is also shown that the correction effect of the method on spectra is general and superior to several other common spectral pre-processing algorithms. The S/S algorithm is mainly used to eliminate multiplicative effects in spectra. Apples, as naturally growing organisms, multiplicative effects caused by differences in physical properties are a significant cause of spectral differences. Therefore, it may be the main reason why this algorithm can effectively improve the prediction performance of the model.

The results of this study demonstrated that the use of DR spectroscopy also has a certain prediction ability for apple whole fruit SSC. In particular, after model fusion, the RPD of DR spectroscopy for the prediction of apple whole fruit SSC was significantly improved. It shows that the model fusion strategy enables the DR spectroscopy to meet the demand for practical detection of apple whole fruit SSC. Due to the simple structure of DR spectroscopy acquisition, the model fusion method can be used to improve the online detection accuracy of apple whole fruit SSC at a low cost.

Not all fusion models improve the predictive performance of models compared to single predictive models. Therefore, exploring the applicable MCMF and CNCMF modeling methods in this study is necessary. The fusion models show some similarities, and all of them are PSO-ELM models with the best enhancement effect. This study fully demonstrates the effectiveness of the proposed model fusion method by building the prediction models of the three mode spectra. It also shows that the boosting effect of the method is not a chance phenomenon.

Compared with other studies on apple SSC online detection, the best prediction model constructed in this study is better than Li et al. (2023) [42], Xia et al. (2019) [16], and Tian et al. (2019) [43], and slightly lower than Chang et al. (2023) [44] and Zheng et al. (2023) [45]. Moreover, the spectrometer used in this study has a lower cost. Therefore, the methods proposed in this study can improve the model prediction performance based on a lower-cost spectrometer.

The spectral pre-processing method proposed in this study enables targeted elimination of multiplicative effects in spectra. The method can be applied in the spectral correction of other agricultural products with significant differences in physical properties. The model fusion methods proposed in this study are different from other previous research methods in that they can fully utilize the contribution of the discarded models and wavelength variables to the overall prediction results and provide new ideas and methods for online detection of apple SSC. However, this study was only conducted for specific varieties of apples, and the applicability to other varieties of apples or other types of fruit needs to be explored in the future. In addition, other quality indicators, such as acidity and moldy heart disease, need to be further explored.

## 4. Conclusions

(1) For the full spectrum, the S/S-PLSR models for all three mode spectra had good prediction performance;
(2) The CARS characteristic wavelength screening algorithm can further improve the prediction performance of the S/S-PLSR models;
(3) The PSO-ELM models of MCMF and CNCMF could simultaneously improve the prediction performance of the three modal spectra for apple whole fruit SSC, so that the DR spectra, which originally had a weaker performance, also had a higher prediction performance;
(4) For the full spectrum, characteristic wavelength, and fusion models, the DR, DT, and FT spectra all had some predictive ability for apple whole fruit SSC, with the FT spectrum having the best predictive ability, followed by the DT spectrum.

The results demonstrate the effectiveness of the proposed spectral correction method and model fusion methods. The proposed methods provide new ideas and approaches to improve the accuracy of online apple quality detection. These methods can be applied to the quality detection of other fruits or agricultural products. The results of the study provide data support for guiding the development of online apple quality detection devices, and they are of great significance and value in reducing the cost of the devices and improving detection accuracy.

## Figures and Tables

**Table 1:** Results of apple SSC statistics.

Acquisition Modes	Dataset	No. of Samples	Max (%)	Min (%)	Average (%)	Standard Deviation (%)
DR	Total	105	16.600	9.000	12.950	1.551
	Calibration	78	16.600	9.000	12.937	1.554
	Prediction	27	16.600	9.100	12.989	1.571
DT	Total	105	16.600	9.000	12.950	1.551
	Calibration	79	16.600	9.000	13.030	1.568
	Prediction	26	15.200	9.000	12.708	1.503
FT	Total	105	16.600	9.000	12.950	1.551
	Calibration	79	16.600	9.000	12.880	1.553
	Prediction	26	16.600	9.000	13.165	1.554

Notes: DR: diffuse reflection, DT: diffuse transmission, FT: full transmission.

**Table 2:** Apple SSC modeling results.

Model	Spectral Mode	Pre-Processing	Factor	Calibration Set		Prediction Set		RPD
				*R*_c_	RMESC (%)	*R*_p_	RMSEP (%)	
PLSR	DR	S/S	LVs: 8	0.877	0.742	0.858	0.811	1.937
	DT	S/S	LVs: 11	0.902	0.641	0.902	0.649	2.316
	FT	S/S	LVs: 10	0.917	0.615	0.912	0.653	2.380
LS-SVR	DR	RAW	γ: 2.592 × 10^4^, $\sigma^{2}$: 1.486 × 10^5^	0.915	0.645	0.890	0.843	1.864
	DT	RAW	γ: 7.565 × 10^5^, $\sigma^{2}$: 4.283 × 10^5^	0.931	0.573	0.893	0.670	2.243
	FT	RAW	γ: 2.272 × 10^5^, $\sigma^{2}$: 2.787 × 10^5^	0.951	0.483	0.910	0.659	2.358
PSO-ELM	DR	SNV	Hidden nodes: 30	0.859	0.791	0.840	0.838	1.875
	DT	SNV	Hidden nodes: 30	0.916	0.626	0.894	0.659	2.281
	FT	SNV	Hidden nodes: 30	0.918	0.612	0.902	0.658	2.362

Notes: PLSR: partial least squares regression, LS-SVR: least squares support vector regression, PSO-ELM: particle swarm optimization–extreme learning machine, DR: diffuse reflection, DT: diffuse transmission, FT: full transmission, S/S: spectra to spectra ratio, RAW: raw spectra, SNV: standard normal variational transform, *R*_c_: the correlation coefficient of calibration, RMSEC: root mean square error of calibration, *R*_p_: the correlation coefficient of prediction, RMSEP: root mean square error of prediction, RPD: relative percentage difference, LVs: latent variables.

**Table 3:** Results of apple SSC modeling based on characteristic wavelengths.

Model	Spectral Mode	Number of Characteristic Wavelengths	LVs	Calibration Set		Prediction Set		RPD
				*R*_c_	RMSEC (%)	*R*_p_	RMSEP (%)	
S/S-CARS-PLSR	DR	45	9	0.899	0.675	0.883	0.749	2.097
	DT	26	8	0.916	0.624	0.907	0.630	2.386
	FT	34	9	0.944	0.508	0.931	0.575	2.703
S/S-BOSS-PLSR	DR	24	6	0.862	0.783	0.857	0.827	1.900
	DT	15	10	0.906	0.658	0.887	0.693	2.169
	FT	24	9	0.938	0.537	0.892	0.696	2.233
S/S-iVISSA-PLSR	DR	481	7	0.902	0.668	0.820	0.909	1.728
	DT	417	8	0.852	0.815	0.810	0.894	1.681
	FT	438	11	0.939	0.531	0.891	0.706	2.201

Notes: S/S: spectra to spectra ratio, PLSR: partial least squares regression, CARS: competitive adaptive reweighted sampling, BOSS: bootstrapping soft shrinkage iVISSA: interval variable iterative space shrinkage approach, DR: diffuse reflection, DT: diffuse transmission, FT: full transmission, LVs: latent variables, *R*_c_: the correlation coefficient of calibration, RMSEC: root mean square error of calibration, *R*_p_ the correlation coefficient of prediction, RMSEP: root mean square error of prediction, RPD: relative percentage difference.

**Table 4:** MCMF modeling results.

Model	Spectral Mode	Factor	Calibration Set		Prediction Set		RPD
			*R*_c_	RMESC (%)	*R*_p_	RMSEP (%)	
SA	DR	(*f*_1_ + *f*_2_ + *f*_3_)/3	0.889	0.712	0.895	0.736	2.135
	DT	(*f*_1_ + *f*_2_ + *f*_3_)/3	0.907	0.658	0.905	0.636	2.363
	FT	(*f*_1_ + *f*_2_ + *f*_3_)/3	0.915	0.629	0.899	0.725	2.143
BG	DR	0.428 *f*_1_ + 0.318 *f*_2_ + 0.254 *f*_3_	0.894	0.695	0.898	0.729	2.155
	DT	0.407 *f*_1_ + 0.355 *f*_2_ + 0.238 *f*_3_	0.911	0.645	0.908	0.622	2.416
	FT	0.547 *f*_1_ + 0.274 *f*_2_ + 0.179 *f*_3_	0.931	0.568	0.915	0.664	2.340
MLR	DR	0.793 *f*_1_ + 0.253 *f*_2_ − 0.025 *f*_3_ −0.266	0.903	0.664	0.888	0.734	2.140
	DT	1.070 *f*_1_ − 0.210 *f*_2_ + 0.157 *f*_3_ −0.217	0.917	0.620	0.911	0.610	2.464
	FT	1.134 *f*_1_ − 0.211 *f*_2_ − 0.075 *f*_3_ +0.026	0.946	0.502	0.936	0.550	2.825
LS-SVR	DR	γ: 6.288, $\sigma^{2}$: 12.348	0.915	0.626	0.881	0.753	2.086
	DT	γ: 28.276, $\sigma^{2}$: 4.662	0.953	0.475	0.898	0.656	2.291
	FT	γ: 38.849, $\sigma^{2}$: 5.462	0.965	0.407	0.940	0.542	2.867
PSO-ELM	DR	Hidden nodes: 15	0.935	0.547	0.931	0.562	2.795
	DT	Hidden nodes: 15	0.944	0.464	0.936	0.518	2.902
	FT	Hidden nodes: 15	0.966	0.401	0.956	0.449	3.461

Notes: SA: simple averaging, BG: Bates–Granger average, MLR: multiple linear regression, LS-SVR: least squares support vector regression, PSO-ELM: particle swarm optimization–extreme learning machine, DR: diffuse reflection, DT: diffuse transmission, FT: full transmission, *R*_c_: the correlation coefficient of calibration, RMSEC: root mean square error of calibration, *R*_p_ the correlation coefficient of prediction, RMSEP: root mean square error of prediction, RPD: relative percentage difference.

**Table 5:** Non-CARS characteristic wavelength modeling results.

Model	Spectral Mode	LVs	Calibration Set		Prediction Set		RPD
			*R*_c_	RMESC (%)	*R*_p_	RMSEP (%)	
PLSR	DR	8	0.874	0.751	0.854	0.820	1.916
	DT	12	0.915	0.629	0.879	0.723	2.079
	FT	10	0.915	0.623	0.909	0.662	2.347

Notes: PLSR: partial least squares regression, DR: diffuse reflection, DT: diffuse transmission, FT: full transmission, *R*_c_: the correlation coefficient of calibration, RMSEC: root mean square error of calibration, *R*_p_ the correlation coefficient of prediction, RMSEP: root mean square error of prediction, RPD: relative percentage difference, LVs: latent variables.

**Table 6:** CNCMF modeling results.

Model	Spectral Mode	Factor	Calibration Set		Prediction Set		RPD
			*R*_c_	RMESC (%)	*R*_p_	RMSEP (%)	
SA	DR	(*f*_4_ + *f*_5_) /2	0.903	0.665	0.891	0.739	2.126
	DT	(*f*_4_ + *f*_5_) /2	0.921	0.607	0.898	0.659	2.281
	FT	(*f*_4_ + *f*_5_) /2	0.936	0.543	0.928	0.593	2.621
BG	DR	0.553 *f*_4_ + 0.447 *f*_5_	0.904	0.661	0.892	0.735	2.137
	DT	0.504 *f*_4_ + 0.496 *f*_5_	0.921	0.607	0.898	0.658	2.284
	FT	0.600 *f*_4_ + 0.400 *f*_5_	0.938	0.532	0.930	0.585	2.656
MLR	DR	0.708 *f*_4_ + 0.321 *f*_5_ − 0.374	0.905	0.656	0.893	0.722	2.176
	DT	0.541 *f*_4_ + 0.470 *f*_5_ − 0.147	0.921	0.607	0.899	0.659	2.281
	FT	1.071 *f*_4_ − 0.072 *f*_5_ + 0.015	0.944	0.508	0.931	0.577	2.693
LS-SVR	DR	γ: 878.253, $\sigma^{2}$: 37.202	0.916	0.618	0.895	0.731	2.149
	DT	γ: 69.051, $\sigma^{2}$: 4.147	0.956	0.455	0.887	0.692	2.172
	FT	γ: 18.710, $\sigma^{2}$: 3.633	0.956	0.455	0.936	0.556	2.795
PSO-ELM	DR	Hidden nodes: 15	0.936	0.546	0.934	0.552	2.846
	DT	Hidden nodes: 15	0.959	0.442	0.944	0.485	3.099
	FT	Hidden nodes: 15	0.958	0.445	0.956	0.445	3.492

Notes: SA: simple averaging, BG: Bates–Granger average, MLR: multiple linear regression, LS-SVR: least squares support vector regression, PSO-ELM: particle swarm optimization–extreme learning machine, DR: diffuse reflection, DT: diffuse transmission, FT: full transmission, *R*_c_: the correlation coefficient of calibration, RMSEC: root mean square error of calibration, *R*_p_ the correlation coefficient of prediction, RMSEP: root mean square error of prediction, RPD: relative percentage difference.

## Data Availability

The original contributions presented in the study are included in the article, further inquiries can be directed to the corresponding author.
